# General and Behavioral Health Screening Under EPSDT for Adolescents in New York Medicaid Managed Care

**DOI:** 10.1001/jamanetworkopen.2026.3060

**Published:** 2026-03-24

**Authors:** Rachel Rosales, David J. Meyers, K. John McConnell, Michael Flores, Emily Feinberg, Elizabeth Burke Bryant, Omar Galárraga

**Affiliations:** 1 Department of Health Services, Policy, and Practice, Brown University School of Public Health, Providence, Rhode Island; 2 Center for Health Systems Effectiveness, Oregon Health & Science University, Portland; 3 Health Equity Research Lab, Cambridge Health Alliance, Cambridge, Massachusetts; 4 Department of Psychiatry, Harvard Medical School, Boston, Massachusetts; 5 Department of Health Services, Policy, and Practice and Hassenfeld Child Health Innovation Institute, Brown University School of Public Health, Providence, Rhode Island; 6 Population Studies and Training Center (PSTC), Brown University, Providence, Rhode Island

## Abstract

**Importance:**

Medicaid-enrolled children are entitled to Early and Periodic Screening, Diagnostic, and Treatment (EPSDT) benefits, which include general and behavioral health (BH) screenings. Adolescents remain among the least likely to receive recommended screenings, and little is known about how screening delivery varies across plans or aligns with plan quality ratings.

**Objectives:**

To examine trends in general and BH screenings under EPSDT among adolescents aged 12 to 18 years in New York Medicaid managed care and to assess whether screening performance differs by managed care organization (MCO) quality ratings.

**Design, Setting, and Participants:**

This cross-sectional study used New York Medicaid administrative data from January 2016 to December 2021, including enrollment and claims from the Transformed Medicaid Statistical Information System analytic files. During the study period, reporting of BH screening was voluntary. The study included adolescents aged 12 to 18 years who were continuously enrolled for at least 6 months in a given calendar year. Data analysis was conducted from March 2025 to January 2026.

**Exposure:**

Enrollment as an adolescent in a Medicaid MCO in New York State.

**Main Outcomes and Measures:**

Claims-based measures of utilization of general and BH screenings consistent with EPSDT services. The primary measures were (1) the percentage of adolescents receiving at least 1 general screening and (2) the percentage receiving at least 1 BH screening during a calendar year. In a supplemental analysis, the correlation between general and BH EPSDT screening rates was tested.

**Results:**

This cross-sectional study included 1 562 342 unique adolescents aged 12 to 18 years enrolled in New York Medicaid from 2016 to 2021 (mean [SD] age, 14.9 [2.0] years; 761 203 [48.7%] female; 7629 [0.5%] American Indian or Alaska Native; 148 576 [9.5%] Asian American or Pacific Islander; 286 003 [18.3%] Black; 204 340 [13.1%] Hispanic; 403 490 [25.8%] White; 512 304 [32.8%] additional groups or missing information). General EPSDT screening rates ranged from 52.5% to 61.0% annually, with the lowest rate observed in 2020 during the onset of the COVID-19 pandemic. BH screening rates increased over time, from 7.7% in 2016 to 21.2% in 2021, but remained substantially lower than general EPSDT screening rates, reaching only about one-third of general screening rates. Screening rates were highest for mid-adolescents (ages 14-16 years) and lowest for ages 12 and 18 years. Across MCOs, general screening rates were relatively consistent, whereas BH screening rates varied widely, with only moderate correlation between the 2 screening types at the plan level (Pearson *r* = 0.47). BH performance showed little alignment with state-assigned MCO quality ratings.

**Conclusions and Relevance:**

In this cross-sectional study of adolescents in New York Medicaid from 2016 to 2021, BH screening rates remained far lower than general EPSDT screening rates, with wide variation across MCOs and limited alignment with state-assigned MCO quality ratings. These gaps may reflect long-standing structural challenges, including previously voluntary reporting of adolescent depression screening by the Centers for Medicare & Medicaid Services under the Mandatory Core Set of Behavioral Health Measures for Medicaid and Children’s Health Insurance Program. Incorporating now-mandatory BH measures into MCO performance benchmarks could increase adolescent BH screening rates, strengthen accountability, support earlier detection of mental health conditions, and reduce variation in preventive care delivery both in New York and nationwide.

## Key Points

**Question:**

How do behavioral health (BH) screening rates compare with general Early and Periodic Screening, Diagnostic, and Treatment (EPSDT) screening rates for adolescents enrolled in New York Medicaid managed care organizations (MCOs)?

**Findings:**

In this cross-sectional study of more than 1.5 million adolescents, BH screening rates under EPSDT were consistently lower than general screening rates, reaching only about one-third the rate of general EPSDT screening. General screening rates were relatively similar across plans over the study period, whereas BH screening rates varied widely and showed little alignment with state-assigned MCO quality ratings.

**Meaning:**

In this cross-sectional study, BH screening for adolescents lagged behind general EPSDT screenings, with wide plan-level variation and poor alignment with state-assigned MCO quality ratings.

## Teaser

This cross-sectional study examines whether adolescents enrolled in Medicaid receive recommended behavioral health screenings under Early and Periodic Screening, Diagnostic, and Treatment (EPSDT) and whether these rates correlate with managed care organization quality ratings.

## Introduction

Early and Periodic Screening, Diagnostic, and Treatment (EPSDT), first enacted in 1967 under the Social Security Act, was a landmark effort to guarantee that Medicaid-enrolled children receive comprehensive preventive care.^1,2^ The benefit entitles these children to “covered services and any other necessary health care, diagnostic services, treatment [for] physical and behavioral health (BH) conditions.”^2,3,4^ Its creation followed evidence of widespread unmet health needs among children in the Head Start program and young adults drafted during the Vietnam War.^1,2^ More than 50 years later, the Centers for Medicare & Medicaid Services (CMS) continues to emphasize compliance, but gaps in screening and treatment for children’s physical and BH needs remain.^1,5^

States share responsibility with the federal government for implementing EPSDT benefits, either directly or through oversight to managed care organizations (MCOs) that administer the benefit.^6^ EPSDT-covered screenings can be performed by any qualified medical professional within the scope of their state-defined practice.^3,6,7^ Most major professional organizations recommend at least 1 annual well-child visit (WCV), which serves as the primary setting for EPSDT delivery.^8,9^ However, adolescents historically have the lowest WCV utilization, a pattern that predates COVID-19 and worsened during the pandemic, with Medicaid-enrolled youth disproportionately impacted compared with peers in commercial plans.^10,11,12^

EPSDT screenings may address medical, behavioral, vision, hearing, and dental health.^3,6^ These screenings are grouped into 2 broad categories: general screenings, which address preventive medical needs like physical examinations, immunizations, vision and hearing checks, and BH screenings, which focus on identifying mental health or substance use disorders.^7,13^ Timely screenings can detect conditions early, reducing complications from co-occurring BH and medical issues and lowering health care costs.^14,15^ Yet, widespread noncompliance with EPSDT requirements has prompted lawsuits in multiple states, underscoring the gap between federal policy and actual implementation.^16^

Addressing this gap is increasingly urgent amid rising rates of adolescent BH disorders. In 2018, 1 in 5 noninstitutionalized youths aged 12 to 17 years experienced a major depressive episode.^17^ In the 2010s, pediatric mental health–related emergency department visits increased from 4.8 million to 7.5 million, with a 5-fold increase in suicide-related visits.^18^ These trends worsened during the COVID-19 pandemic, driven by factors including social isolation, school disruptions, and health concerns.^19^ By 2021, 41% of US adolescents reported persistent feelings of sadness, hopelessness, stress, and anxiety,^20,21^ and rates of suicidal ideation in adolescents nearly doubled during the pandemic.^20,22^

Despite these escalating BH needs, delivery of EPSDT screenings has not kept pace. Nationally, just under 60% of children received at least 1 EPSDT screening.^3^ This gap is larger for BH screenings, which are less consistently implemented or tracked.^23,24^ In response, the Center for Medicaid and CHIP (Children’s Health Insurance Program) Services issued an informational bulletin in August 2022 reminding state health officials that conducting BH screenings and ensuring access to BH treatment are required components of EPSDT.^25^ This directive is especially relevant for adolescents, who (1) are at higher risk for missed screening opportunities because they are less likely to attend annual WCVs and (2) are at heightened risk for BH conditions emerging during this period.^26,27^

State approaches to EPSDT implementation vary widely. New York’s Medicaid program, one of the nation’s largest and most diverse, mirrors national Medicaid patterns, in which Black, Hispanic, and Indigenous children are disproportionately represented.^28^ This demographic profile makes New York an appropriate case study for examining how effectively EPSDT supports adolescent BH screening alongside general preventive care. Nearly all children enrolled in New York Medicaid (94.6% according to MACPAC) are in MCOs, through which the state oversees the administration of EPSDT benefits.^29^ Patterns of general and BH screening delivery across MCOs are not well characterized, and national reports typically summarize only aggregate compliance, lacking the granularity needed to pinpoint gaps. This study provides a descriptive assessment of adolescent BH and general EPSDT screening rates from January 2016 to December 2021, with January 2020 to December 2021 patterns interpreted in the context of COVID-19–related disruptions to preventive services.

## Methods

### Study Design and Setting

This cross-sectional study used New York State Medicaid administrative data from the Transformed Medicaid Statistical Information System analytic files (TAF), including enrollment and claims data from 2016 to 2021. We examined trends in general and BH EPSDT screening rates among adolescents, disaggregated by age, MCO, and MCO quality rating.

The study protocol was approved by the Brown University institutional review board as exempt research and received a waiver of informed consent because the study was a secondary analysis of deidentified data. The study followed the Strengthening the Reporting of Observational Studies in Epidemiology (STROBE) reporting guideline for cross-sectional studies.

We excluded enrollees who were dually eligible for Medicare and Medicaid due to incomplete claims capture; enrolled in emergency Medicaid, as they are ineligible for full EPSDT benefits; or in foster care due to separate service arrangements and coverage pathways, such as enhanced EPSDT services with care coordination and case management, targeted BH and child welfare engagement, and specialized screenings for children in foster care.^30^ Enrollee–calendar years with fewer than 6 months of continuous enrollment were also excluded. A flowchart of inclusion and exclusion criteria is provided in eFigure 1 in Supplement 1.

Each adolescent could contribute multiple enrollee–calendar years if continuously enrolled. For descriptive purposes, we report the number of unique adolescents included in the study. Race and ethnicity were derived from TAF-reported data, with individuals categorized as belonging to additional groups or having missing information if classified as multiracial, non-Hispanic; other, non-Hispanic; or if race and ethnicity data were missing. Enrollee-year observations missing MCO plan identification were included in statewide estimates but excluded from analyses requiring plan attribution.

Plan quality ratings were obtained from the New York State Department of Health (NYSDOH). These ratings are derived from the Quality Assurance Reporting Requirements (QARR) system, which evaluates MCOs based on a comprehensive set of performance measures and is independently validated.^31^ The QARR system is designed to inform consumers about the quality of care provided by MCOs, including access to services, preventive care, and the medical management of select conditions.^31^ Although the underlying QARR performance indicators are publicly available, the star ratings for the years used in this study are not publicly posted and were provided by NYSDOH upon request. The QARR Behavioral Health for Children and Adolescents domain includes measures such as metabolic monitoring for children on antipsychotics, use of first-line psychosocial care for new antipsychotic prescriptions, and follow-up care for children newly prescribed attention-deficit/hyperactivity disorder medication.^32^ Because our study assesses preventive BH screening among all adolescents, we instead used ratings for the Child and Adolescent Care domain, which encompass measures of preventive care, immunizations, and WCVs, in this analysis.^33^ Annual ratings were aligned with the corresponding calendar year of Medicaid claims and enrollment data. MCO plan identifiers were obtained from the Office of Health Insurance Programs, and MCO service regions were taken from the NYSDOH’s Managed Care Regional Consumer Guides to assign plans to broader geographic regions for descriptive context.^34^ Additional details regarding enrollment numbers, quality ratings, and regions served are provided in eMethods 2 in Supplement 1.

### Outcome Variables

The primary outcomes of this study were the rates of general and BH EPSDT screenings among adolescents enrolled in New York State Medicaid from 2016 to 2021. Screening measures were defined according to CMS form CMS-416 reporting instructions and NYSDOH guidance, specifically the Children and Family Treatment and Support Services Provider Manual under EPSDT protocols.^35,36^

General EPSDT screenings included comprehensive health assessments intended to identify physical health needs, developmental milestones, and preventive health measures. These were identified using a combination of *Common Procedural Terminology* (*CPT*)/Healthcare Common Procedure Coding System (HCPCS) procedure codes and *International Statistical Classification of Diseases and Related Health Problems, Tenth Revision *(*ICD-10*) diagnosis codes consistent with CMS guidance for EPSDT service reporting.

BH EPSDT screenings included standardized behavioral screening tools recommended for early identification of BH conditions. These screenings were captured via procedure codes representing BH screening services and relevant diagnostic codes indicating screening encounters or assessments conducted under EPSDT benefit provisions.

As a secondary descriptive measure, we identified annual WCVs using relevant *CPT*/HCPCS codes. Because WCVs are the primary clinical encounter during which EPSDT screenings are expected to occur, reporting WCV receipt provides context for whether low BH screening rates reflect limited visit attendance, missed screening opportunities during visits, or both. Additional details about procedure, diagnosis, and program type codes utilized to identify screening types are provided in eMethods 1 in Supplement 1.

### Statistical Analysis

Screening rates were calculated as the percentage of eligible adolescents who received at least 1 general or BH EPSDT screening within each calendar year. Because the analysis includes the full population of Medicaid-enrolled adolescents, rather than a sampled subset, population-level descriptive reporting provides the most direct and interpretable summary of EPSDT performance. We stratified screening rates by age, MCO, and MCO quality star rating as assigned by NYSDOH.

In our supplemental analysis, we examined the association between general and BH EPSDT screening rates at the MCO level, assessing whether plans with higher general screening rates were associated with greater BH screening uptake at the plan level. These visualizations and additional detail are provided in eFigure 2 in Supplement 1. All analyses were conducted between March 2025 and January 2026 using Stata version 18 (StataCorp) and SAS version 9.4 (SAS Institute).

## Results

### Sample Population

The study sample included 1 562 342 adolescents aged 12 to 18 years in New York Medicaid from 2016 to 2021 (mean [SD] age, 14.9 [2.0] years; 761 203 [48.7%] female; 7629 [0.5%] American Indian or Alaska Native; 148 576 [9.5%] Asian American or Pacific Islander; 286 003 [18.3%] Black; 204 340 [13.1%] Hispanic; 403 490 [25.8%] White; 512 304 [32.8%] additional groups or missing information). Each adolescent could contribute multiple enrollee-years if continuously enrolled, with a total of 4 515 521 enrollee-years across 2016 to 2021. The Table summarizes the enrollee demographic distribution of the sample by EPSDT screening status, categorized into 4 mutually exclusive groups: (1) no general or BH screening, (2) BH screening only, (3) general screening only, and (4) both general and BH screenings.

**Table.** Summary Statistics by General and Behavioral Health EPSDT Screening

Characteristic	Population in each EPSDT screening type group, No. (%)^a^			
	No general or BH	BH only	General only	Both general and BH
Age, y				
12	359 149 (56.7)	888 (0.1)	208 767 (32.9)	64 888 (9.2)
13	207 064 (33.7)	1054 (0.2)	300 361 (48.9)	106 362 (15.1)
14	200 744 (33.5)	1028 (0.2)	287 677 (48.0)	109 958 (15.6)
15	203 478 (34.8)	1105 (0.2)	274 251 (46.9)	106 123 (15.1)
16	198 599 (34.5)	1077 (0.2)	270 336 (47.0)	105 192 (14.9)
17	197 133 (34.8)	1216 (0.2)	266 515 (47.1)	101 254 (14.4)
18	526 568 (55.9)	1663 (0.2)	302 833 (32.2)	110 238 (11.7)
Sex				
Female	877 666 (40.0)	4612 (0.2)	946 696 (43.1)	365 691 (16.7)
Male	1 015 069 (43.7)	3419 (0.1)	964 044 (41.5)	338 324 (14.6)
Race and ethnicity				
American Indian or Alaska Native	10 881 (48.1)	45 (0.2)	8631 (38.1)	3073 (13.6)
Asian American or Pacific Islander	144 038 (33.1)	421 (0.1)	233 318 (53.6)	57 198 (13.1)
Black	391 313 (47.5)	1919 (0.2)	312 233 (37.9)	118 563 (14.4)
Hispanic	243 689 (37.6)	1735 (0.3)	292 931 (45.2)	109 304 (16.9)
White	511 401 (44.6)	1572 (0.1)	449 299 (39.2)	183 764 (16.0)
Additional groups or missing information^b^	591 413 (41.1)	2339 (0.2)	614 328 (42.7)	232 113 (16.1)

Abbreviations: EPSDT, Early and Periodic Screening, Diagnostic, and Treatment; BH, Behavioral Health.

^a^ The analysis includes all adolescents (1 562 342 unique adolescents) continuously enrolled in New York Medicaid for at least 6 months. Percentages reflect the proportion of individuals within each demographic subgroup (row) who received general or BH EPSDT screenings. For example, among 12-year-olds, 56.7% received neither type of screening.

^b^ Individuals categorized as belonging to additional groups or having missing information include those classified as multiracial, non-Hispanic; other, non-Hispanic; or those with missing race and ethnicity values in the Transformed Medicaid Statistical Information System annual demographic and eligibility analytic files.

Receipt of EPSDT screenings varied modestly by sex and race and ethnicity (Table). Female adolescents received screenings more often than male adolescents, and across all racial and ethnic groups, receipt of both screening types remained far lower than receipt of general EPSDT screenings alone.

### Trends in EPSDT Screening Over Time

**Figure 1.**
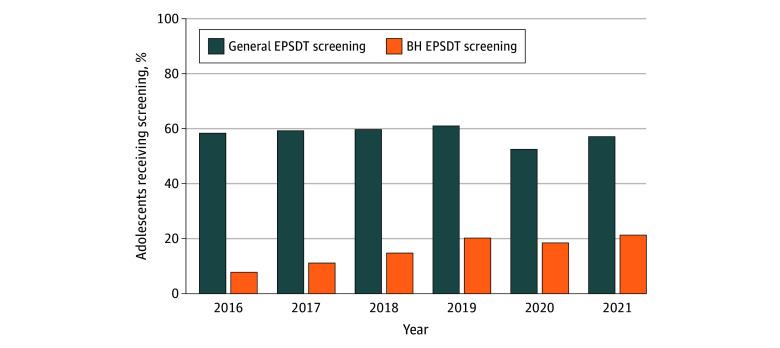
Bar Graphs of the Percentage of Medicaid-Enrolled Adolescents Ages 12 to 18 Years Who Received General and Behavioral Health (BH) Early and Periodic Screening, Diagnostic, and Treatment (EPSDT) Screenings, 2016-2021 This figure presents the annual percentage of adolescents ages 12 to 18 years enrolled in New York Medicaid who received at least 1 general EPSDT screening (including preventive visits and physical examinations) or BH EPSDT screening (including standardized BH assessment or psychiatric diagnostic evaluation) during the calendar year. The analysis includes all adolescents (1 562 342 unique adolescents) continuously enrolled in New York Medicaid for at least 6 months.

From 2016 to 2019, general EPSDT screening rates were relatively stable (58.4% [419 248 of 718 429] to 61.0% [444 823 of 728 935]), declined to 52.5% (406 240 of 773 827) in 2020, and rebounded to 57.1% (476 761 of 835 262) in 2021 (Figure 1). In contrast, BH screening rates were much lower but increased steadily throughout the study period, from 7.7% (55 479) in 2016 to 21.2% (177 297) in 2021. WCV rates were moderate (eg, 47.9% [344 057] in 2016; 48.2% [402 253] in 2021), indicating that many adolescents attended preventive visits during which EPSDT screenings could have occurred.

**Figure 2.**
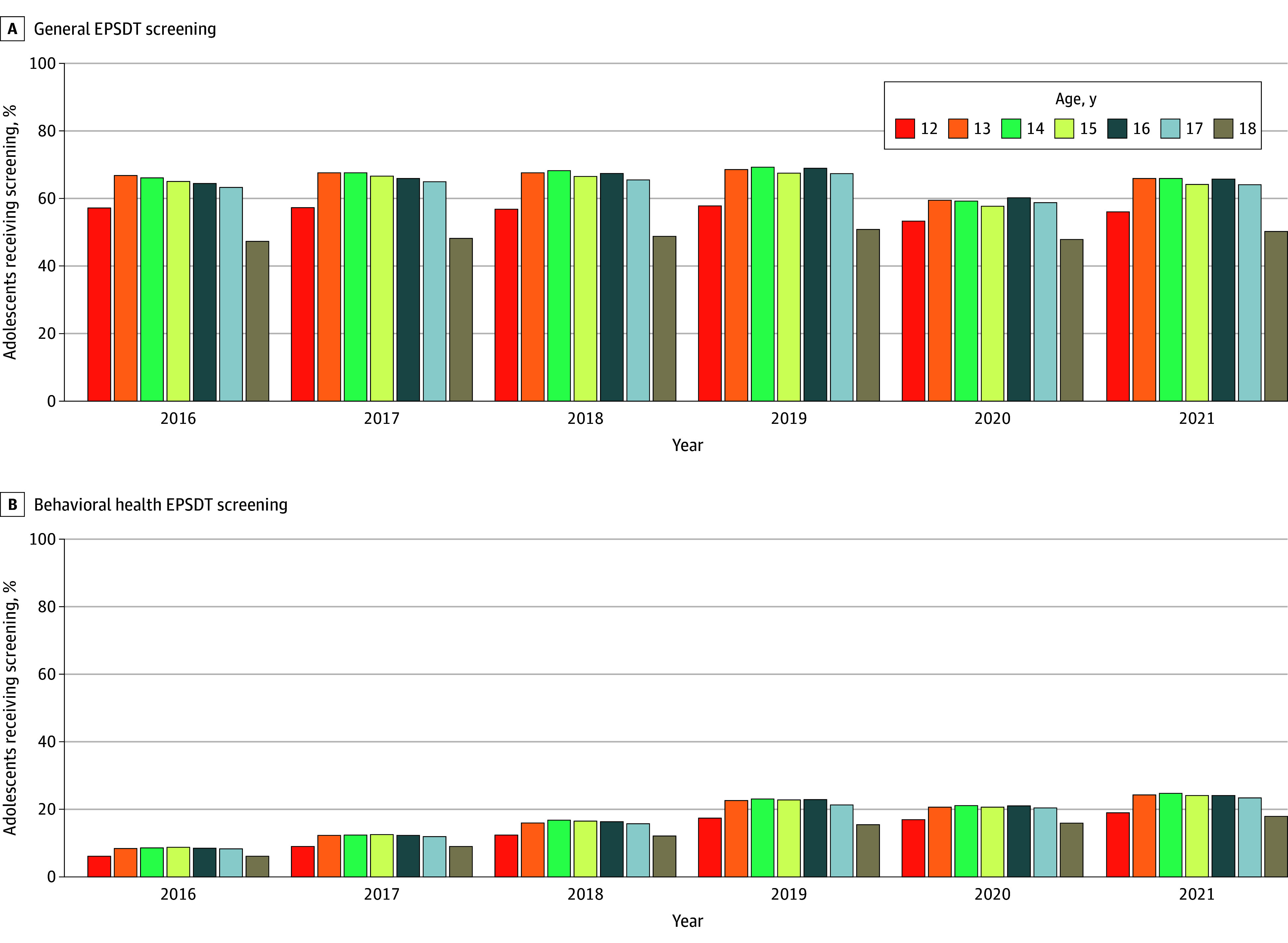
Bar Graphs of the Annual Percentage of Adolescents Ages 12 to 18 Years Enrolled in New York Medicaid Who Received at Least 1 General or Behavioral Health Early and Periodic Screening, Diagnostic, and Treatment (EPSDT) Screening, by Age and Calendar Year, 2016-2021 Each panel displays the age-specific percentage of adolescents receiving at least 1 screening during the calendar year, stratified by age at the time of screening. The analysis includes all adolescents (1 562 342 unique adolescents) continuously enrolled in New York Medicaid for at least 6 months.

Screening rates varied by age (Figure 2). General EPSDT screenings peaked across ages in 2019, before the onset of the COVID-19 pandemic. Rates increased through mid-adolescence, with 14- to 16-year-olds demonstrating the highest uptake. Rates were consistently lowest at ages 12 and 18 years. In 2019, 69.3% of those aged 14 years (66 669 of 96 199) received general EPSDT screenings compared with 57.9% of those aged 12 years (80 363 of 138 882) and 50.8% of those aged 18 years (41 209 of 81 106). Screening rates declined across all ages in 2020 and rebounded in 2021, although not to 2019 levels.

BH screenings exhibited a similar overall age pattern, lowest at ages 12 and 18 and highest in mid-adolescence, but were more attenuated than general EPSDT screenings. In 2019, 23.0% of those aged 14 years (22 152 of 96 199) received BH screening, compared with 17.4% of those aged 12 years (24 200 of 138 882) and 15.4% of those aged 18 years (12 493 of 81 106). Unlike general screenings, BH screenings rates continued to rise through 2021 despite the 2020 decline, ultimately exceeding their 2019 levels.

### Variation by Managed Care Organization

**Figure 3.**
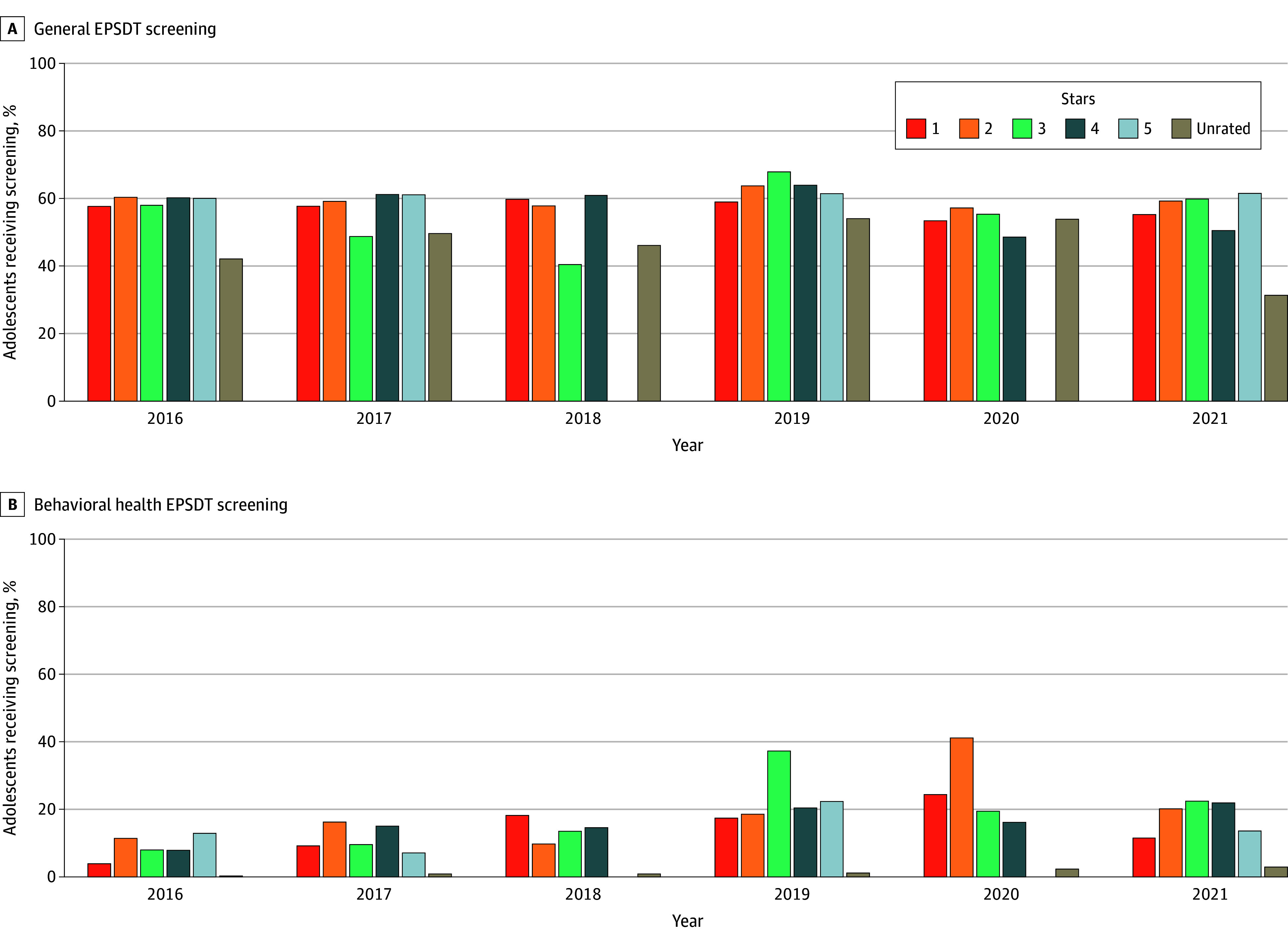
Bar Graphs of the Annual Percentage of Adolescents Ages 12 to 18 Years Enrolled in New York Medicaid Who Received at Least 1 General or Behavioral Health Early and Periodic Screening, Diagnostic, and Treatment (EPSDT) Screening, by Medicaid Managed Care Organization Quality Rating and Calendar Year, 2016-2021 Each panel displays the age-specific percentage of adolescents receiving at least 1 screening during the calendar year, stratified by plan rating as assigned by the New York State Department of Health. The analysis includes all adolescents (1 562 342 unique adolescents) continuously enrolled in New York Medicaid for at least 6 months. There were no 5-star rated plans in the data for 2018 and 2020.

Figure 3 shows general and BH screening rates by NYSDOH-assigned MCO quality rating. Across all ratings, BH screening rates were consistently lower than general EPSDT rates, and screening performance did not show a consistent pattern by quality rating.

**Figure 4.**
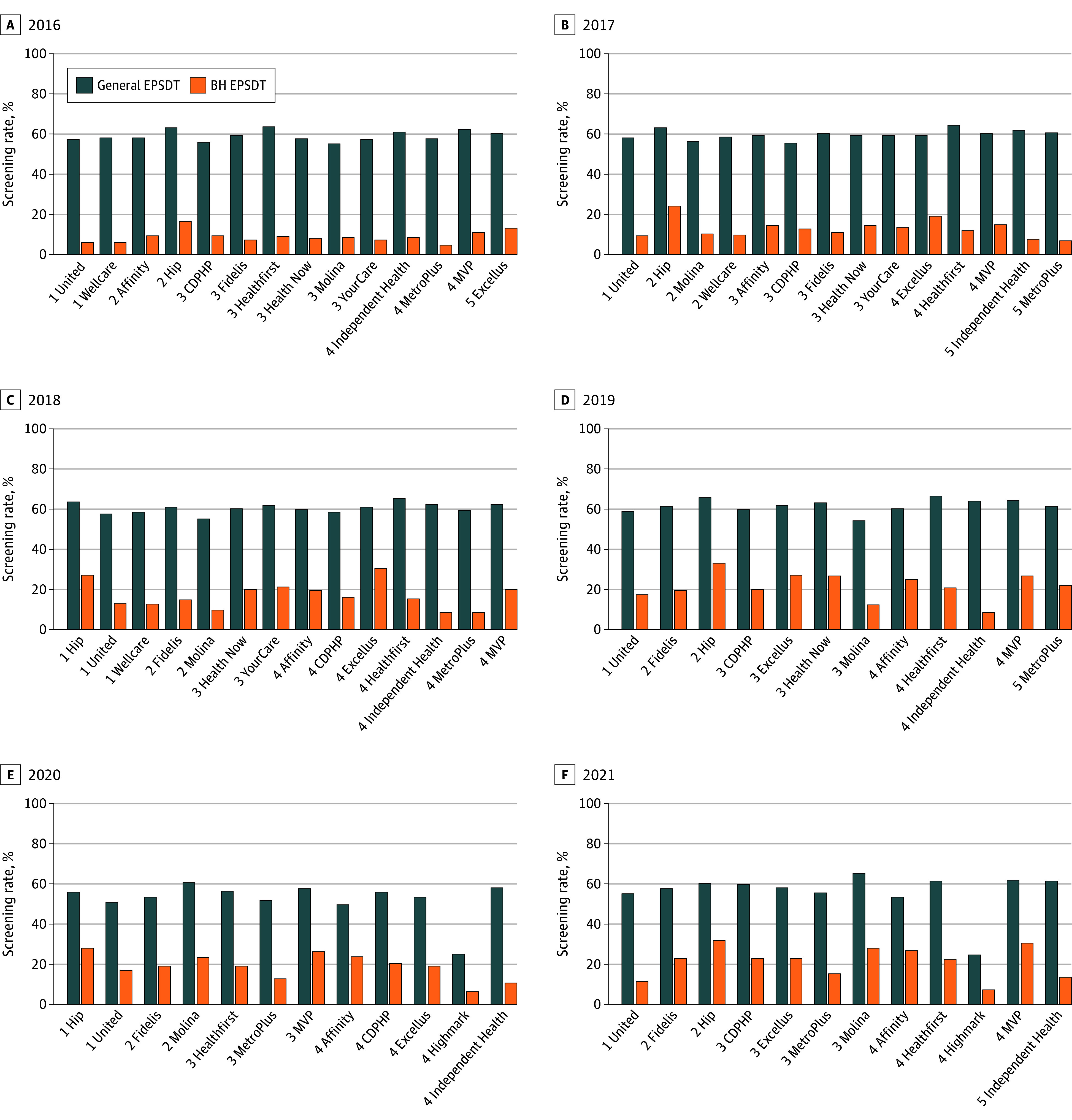
Bar Graphs of General and Behavioral Health (BH) Early and Periodic Screening, Diagnostic, and Treatment (EPSDT) Screening Rates by Medicaid Managed Care Organization and Year, Stratified by Quality Rating, 2016-2021 This figure displays annual screening rates for general EPSDT services and BH EPSDT services among adolescents aged 12 to 18 years, disaggregated by Medicaid managed care organization (MCO) in New York State from 2016 to 2021. Each panel represents a different year, with MCOs stratified by their Medicaid quality rating (1-5 stars, as indicated at the beginning of each plan name) as assigned by the New York State Department of Health.

Figure 4 presents screening rates at the MCO plan level. General EPSDT screening rates were relatively uniform across plans, whereas BH screening rates were substantially lower and highly variable, with some plans performing nearly twice as well as others. Across all plans, BH screening rates remained well below general screening rates, and plan-level performance showed no consistent association with state-assigned quality ratings.

The association between general and BH screening rates by MCO and year is displayed in eFigure 2 in Supplement 1. General and BH screening rates were moderately correlated at the plan level (Pearson *r* = 0.47). Local polynomial smooth plots and scatterplots illustrating this association, along with additional detail, are provided in eFigure 2 in Supplement 1.

## Discussion

This study of New York’s delivery of EPSDT to adolescents has 3 key findings. First, BH screenings increased from 7.7% in 2016 to 21.2% in 2021, but remained far less common than general EPSDT screenings, which were received by approximately 60% of adolescents annually. Moderate WCV rates suggest that gaps in BH screening are not solely explained by low WCV attendance. Second, screening rates varied by age, with mid-adolescents (ages 14-16 years) more often receiving both general and BH screenings, highlighting opportunities to improve delivery for younger and older adolescents. Third, screening rates differed across Medicaid MCOs, with BH screenings showing particularly wide plan-level variation and only moderate correlation with general screening rates.

These findings align with prior research documenting persistently low adolescent BH screening rates nationally, despite recommendations for annual, standardized BH assessments.^17,37^ Our study extends this literature with an EPSDT-specific analysis and by examining plan-level variation, an area with limited evidence.

The trends observed in this study reflect both progress and persistent gaps. General EPSDT screening rates remained relatively stable over time, aside from a decline in 2020 during the onset of the COVID-19 pandemic, while BH screening rates more than doubled. The continued rise in BH screening rates is notable given broader declines in BH treatment initiation and WCVs during the pandemic.^12,38,39,40^ This increase may instead reflect heightened attention to adolescent mental health amid social isolation, school disruptions, and pandemic-related stressors.^19^ Continuous Medicaid coverage under the Families First Coronavirus Response Act also protected adolescents’ access to preventive care during a period of service disruptions.^41^ Yet, BH screenings continued to lag substantially behind general EPSDT screenings, with even the highest BH rates reaching only one-third of the general screening rate. This is concerning given adolescence is a high-risk period for emerging BH conditions and missed opportunities for early detection may interfere with timely intervention.

At the plan level, BH screening rates varied widely across MCOs, whereas general screening rates were relatively consistent. The moderate correlation between general and BH screening rates suggests that some plans may deliver more coordinated preventive care. However, state-assigned quality ratings did not align with BH screening performance. This misalignment reflects 2 structural issues. First, New York’s quality rating system has historically not incorporated clinically focused BH performance metrics, in contrast to Healthcare Effectiveness Data and Information Set (HEDIS) measures developed by the National Committee for Quality Assurance, such as the percentage of adolescents with depression who have a documented 9-item Patient Health Questionnaire score.^42,43^ Such measures capture whether plans are systematically identifying depressive symptoms, an approach not reflected in state quality ratings during the study period. Second, until 2024, CMS did not require mandatory reporting of adolescent depression screening under the Core Set of Behavioral Health Measures, even though EPSDT guidance emphasized that BH screenings were included.^44,45^ As a result, MCOs may not have prioritized BH screening in ways that aligned with NYSDOH plan ratings, and higher-rated plans were not necessarily performing well on adolescent BH measures that were voluntary at the time. With CMS now mandating these measures, attention to adolescent BH screening is expected to increase nationally and within New York, potentially improving plan alignment with both preventive care goals and state quality ratings.

An additional consideration is that MCOs in New York do not operate uniformly across the state. Several plans (eg, United) serve multiple regions, while others are geographically concentrated (eg, CDPHP). Although differences in screening rates may partly reflect regional variation in clinician supply or health system resources, adolescent BH screenings remained low relative to each plan’s general EPSDT screenings, even among multiregion plans. This suggests that low uptake is not solely driven by geography and may reflect plan-level factors and broader system challenges.

This study contributes to the literature by providing a granular, state-specific analysis of EPSDT delivery among adolescents. Unlike prior reports that present only aggregate screening rates, we document plan-level variation in BH screening and a disconnect between state-assigned MCO quality ratings and BH screening performance. New York’s recently approved 1115 waiver amendment, which expands investments in BH reimbursement, primary care–BH integration, and health-related social supports, may help strengthen screening infrastructure, increase uptake, and reduce the plan-level variation observed in our study.^46^

### Limitations

This study had several limitations. First, this is an associational analysis, and the observed trends should not be interpreted as causal. Second, EPSDT BH screenings are historically underreported in claims data, which given that Medicaid payments are claims-based, has important implications for informing BH policy and program design. Third, we required at least 6 months of continuous enrollment in a calendar year to balance sample size and the ability to capture service use. While a longer enrollment period might better capture children’s eligibility for annual WCVs, it would restrict the sample and potentially reduce representativeness. This limitation is unlikely to account for the substantial difference between general and BH screening rates, which remains the central finding of this study. Additionally, differences in how data sources define reporting periods (eg, fiscal year vs calendar year, as used in this study) could affect comparability across reports. Despite these limitations, to our knowledge, this is the first study to provide a granular, state-level analysis of adolescent EPSDT screening trends that highlights gaps and opportunities for improving preventive BH care.

## Conclusions

In this cross-sectional study of more than 1.5 million adolescents ages 12 to 18 years enrolled in New York Medicaid from 2016 to 2021, BH screening rates increased over time but remained substantially lower than general EPSDT screenings, reaching only about one-third of general screening rates. General screening rates were consistent across MCOs, whereas BH screening rates varied widely and showed little alignment with state quality ratings, which during the study period did not incorporate adolescent BH screening performance despite adolescence being a high-risk period for emerging BH conditions. These findings underscore opportunities for Medicaid programs to strengthen preventive BH care by more fully integrating BH screening measures into managed care oversight and performance benchmarking to improve accountability. CMS implemented mandatory reporting of adolescent depression screening as part of the Medicaid and CHIP Behavioral Health Core Set beginning in 2024. These initiatives are likely to positively impact both performance and reporting of BH screening in New York and nationally.

## Notes

Supplement 1. **eFigure 1.** Inclusion and Exclusion Criteria for Study Sample **eMethods 1.** Screening Type Measure Definitions **eMethods 2.** Characteristics of Medicaid Managed Care Organizations (MCOs) Serving Adolescents in New York State, 2016 to 2021 **eFigure 2.** Association Between General and Behavioral Health (BH) EPSDT Screening Rates Across Medicaid Managed Care Organizations (MCOs)

Supplement 2.
Data Sharing Statement
