# Supplementary material for: General and Behavioral Health Screening Under EPSDT for Adolescents in New York Medicaid Managed Care
Source: JAMA Netw Open. 2026 Mar 24;9(3):e263060. doi: 10.1001/jamanetworkopen.2026.3060 (PMC13014173; doi:10.1001/jamanetworkopen.2026.3060)
Supplement: Supplement 1. — eFigure 1. Inclusion and Exclusion Criteria for Study Sample eMethods 1. Screening Type Measure Definitions eMethods 2. Characteristics of Medicaid Managed Care Organizations (MCOs) Serving Adolescents in New York State, 2016 to 2021 eFigure 2. Association Between General and Behavioral Health (BH) EPSDT Screening Rates Across Medicaid Managed Care Organizations (MCOs) [file jamanetwopen-e263060-s001.pdf]

Supplemental Online Content

Rosales R, Meyers DJ, McConnell KJ, et al. General and behavioral health screening under EPSDT for adolescents in New York Medicaid managed care. *JAMA Netw Open.* 2026;9(3):e263060. doi:10.1001/jamanetworkopen.2026.3060

eFigure 1. Inclusion and Exclusion Criteria for Study Sample

eMethods 1. Screening Type Measure Definitions

eMethods 2. Characteristics of Medicaid Managed Care Organizations (MCOs) Serving Adolescents in New York State, 2016 to 2021

eFigure 2. Association Between General and Behavioral Health (BH) EPSDT Screening Rates Across Medicaid Managed Care Organizations (MCOs)

This supplemental material has been provided by the authors to give readers additional information about their work.

© 2026 Rosales R et al. *JAMA Network Open*.

eFigure 1. Inclusion and Exclusion Criteria for Study Sample

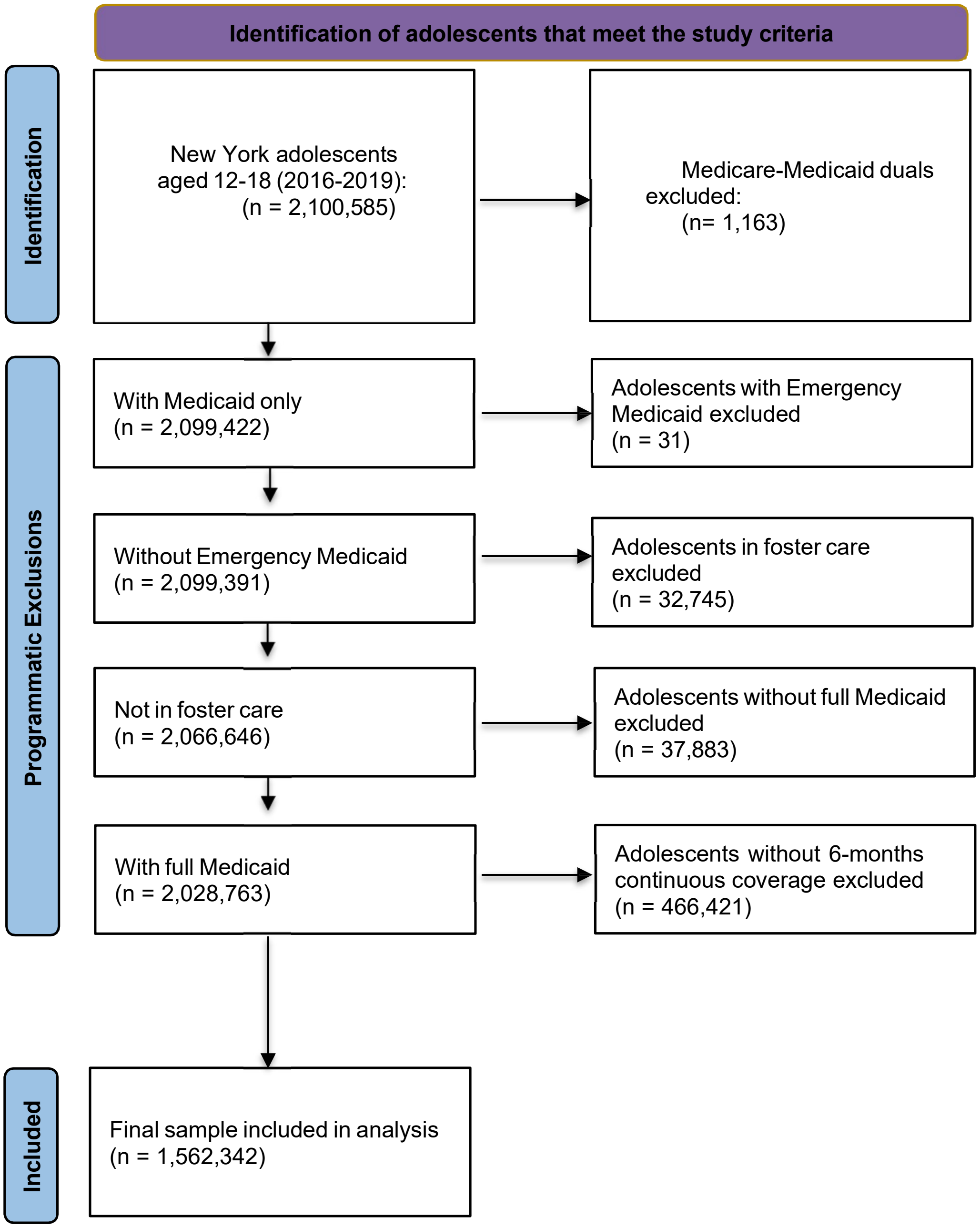

This figure illustrates the stepwise process for defining the analytic cohort of adolescents aged 12–18 years enrolled in New York Medicaid from 2016–2021. Exclusions included enrollees dually eligible for Medicare and Medicaid, those enrolled in Emergency Medicaid, adolescents in

© 2026 Rosales R et al. *JAMA Network Open*.

foster care, and enrollee-calendar years with fewer than six months of continuous enrollment. The final cohort includes adolescents meeting all inclusion criteria.

© 2026 Rosales R et al. *JAMA Network Open*.

eMethods 1. Screening Type Measure Definitions

Individuals were identified as having received general or behavioral health (BH) Early and Periodic Screening, Diagnostic and Treatment (EPSDT) screenings based on specific procedure (CPT/HCPCS) and diagnosis (ICD-10) codes, as well as program type indicators in accordance with CMS Form CMS-416 reporting instructions and New York State Department of Health guidance, specifically the Children and Family Treatment and Support Services Provider Manual under EPSDT protocols.[34,35] The codes used to classify each type of screening are summarized in the table below:

Screening Type	Code Type	Codes/ Notes
General EPSDT	CPT/HCPCS (a)	96110, 99401, 99402, 99403, 99404, 99411, 99412, 99383–99385, 99393–99395, 99429, T1015
General EPSDT	ICD-10 (b)	Z0000, Z0001, Z00121, Z00129, Z1389, Z139, Z1341, Z1342, Z762
Behavioral Health	CPT/HCPCS	96160, 96127, 90791, 90792, H0031
Behavioral Health	ICD-10	Z1330, Z1331, Z1339, Z1340, Z1341, Z1389, Z139, Z1332, Z6221, Z62810–Z62822, Z6372
Behavioral Health	CPT/HCPCS + BH Z-codes (c)	99383–99385, 99393–99395, 96110 (used if a BH Z-code is present) (d)
Any EPSDT	Program Type	1 (indicates EPSDT program) (e)
Well-Child Visit	CPT /HCPCS	99384, 99385, 99394, 99395

Notes:

a. CPT = Current Procedural Terminology; HCPCS = Healthcare Common Procedure Coding System.
b. ICD-10 = International Classification of Diseases, 10th Revision.
c. Some CPT/HCPCS codes used to identify BH screenings overlap with general EPSDT but are counted as BH if linked to a BH diagnosis or Z-code.

© 2026 Rosales R et al. *JAMA Network Open*.

d. Certain CPT codes (99383–99385, 99393–99395) are only classified as BH screenings when a behavioral health–related Z-code is also present.
e. Program type code “1” indicates the service is delivered under the EPSDT program.

© 2026 Rosales R et al. *JAMA Network Open*.

eMethods 2. Characteristics of Medicaid Managed Care Organizations (MCOs) Serving Adolescents in New York State, 2016 to 2021

This table summarizes key features of Medicaid MCOs included in the analysis, including:

(a) the regions in which each plan operates obtained from the Managed Care Regional Consumer Guides by the New York State Department of Health (NYSDOH)[33],
(b) the number of eligible adolescents enrolled obtained from the Medicaid TAF enrollment files,
(c) and the quality ratings assigned by the New York State Department of Health. Although the underlying QARR performance indicators are publicly available, the star ratings for the years used in this study are not publicly posted and were provided to us by NYSDOH upon request. The QARR "Behavioral Health for Children and Adolescents" domain includes measures such as metabolic monitoring for children on antipsychotics, use of first-line psychosocial care for new antipsychotic prescriptions, and follow-up care for children newly prescribed ADHD medication.[47] Because our study assesses preventive BH screening among all adolescents, we instead used ratings for the "Child and Adolescent Care" domain, which encompass measures of preventive care, immunizations, and WCVs, were used in this analysis.[32] Each MCO's star rating corresponds to the specific calendar year of analysis rather than an aggregate or summary value across years.

Year	Managed Care Organization	Regions Served (a)	Number of Eligible Adolescents Enrolled (b)	Quality Rating by NYSDOH (c)
2016	AFFINITY	Hudson Valley, Long Island, New York City	34,480	2
2017	AFFINITY	Hudson Valley, Long Island, New York City	33,992	3
2018	AFFINITY	Hudson Valley, Long Island, New York City	32,993	4
2019	AFFINITY	Hudson Valley, Long Island, New York City	31,065	4
2020	AFFINITY	Hudson Valley, Long Island, New York City	33,834	4
2021	AFFINITY	Hudson Valley, Long Island, New York City	33,795	4
2016	AMIDA	New York City	33	N/A
2017	AMIDA	New York City	47	N/A
2018	AMIDA	New York City	54	N/A
2019	AMIDA	New York City	49	N/A

© 2026 Rosales R et al. *JAMA Network Open*.

Year	Managed Care Organization	Regions Served	Number of Eligible Adolescents Enrolled	Quality Rating by NYSDOH
2020	AMIDA	New York City	59	N/A
2021	AMIDA	New York City	69	N/A
2016	CDPHP	Northeast	15,019	3
2017	CDPHP	Northeast	13,949	3
2018	CDPHP	Northeast	13,783	4
2019	CDPHP	Northeast	13,807	3
2020	CDPHP	Northeast	15,024	4
2021	CDPHP	Northeast	16,627	3
2016	EXCELLUS	Central, Northeast, Western	28,981	5
2017	EXCELLUS	Central, Northeast, Western	28,434	4
2018	EXCELLUS	Central, Northeast, Western	29,299	4
2019	EXCELLUS	Central, Northeast, Western	29,308	3
2020	EXCELLUS	Central, Northeast, Western	32,131	4
2021	EXCELLUS	Central, Northeast, Western	35,656	3
2016	FIDELIS	Central, Hudson Valley, Long Island, Northeast, New York City, Western	170,832	3
2017	FIDELIS	Central, Hudson Valley, Long Island, Northeast, New York City, Western	179,668	3
2018	FIDELIS	Central, Hudson Valley, Long Island, Northeast, New York City, Western	188,312	2
2019	FIDELIS	Central, Hudson Valley, Long Island, Northeast, New York City, Western	193,783	2
2020	FIDELIS	Central, Hudson Valley, Long Island, Northeast, New York City, Western	227,197	2
2021	FIDELIS	Central, Hudson Valley, Long Island, Northeast, New York City, Western	249,984	2
2016	HEALTHFIRST	Hudson Valley, Long Island, New York City	135,313	3
2017	HEALTHFIRST	Hudson Valley, Long Island, New York City	140,147	4

© 2026 Rosales R et al. *JAMA Network Open*.

Year	Managed Care Organization	Regions Served	Number of Eligible Adolescents Enrolled	Quality Rating by NYSDOH
2018	HEALTHFIRST	Hudson Valley, Long Island, New York City	146,315	4
2019	HEALTHFIRST	Hudson Valley, Long Island, New York City	147,890	4
2020	HEALTHFIRST	Hudson Valley, Long Island, New York City	162,554	3
2021	HEALTHFIRST	Hudson Valley, Long Island, New York City	177,157	4
2016	HEALTHNOW	Hudson Valley, Long Island, New York City	4,576	3
2017	HEALTHNOW	Hudson Valley, Long Island, New York City	4,661	3
2018	HEALTHNOW	Hudson Valley, Long Island, New York City	5,076	3
2019	HEALTHNOW	Hudson Valley, Long Island, New York City	5,124	3
2020	HEALTHNOW	Hudson Valley, Long Island, New York City	5,551	N/A
2021	HEALTHNOW	Hudson Valley, Long Island, New York City	6,571	N/A
2020	HIGHMARK	Western	9,219	4
2021	HIGHMARK	Western	8,861	4
2016	HIP (EMBLEMHEALTH)	Hudson Valley, Long Island, New York City	27,348	2
2017	HIP (EMBLEMHEALTH)	Hudson Valley, Long Island, New York City	23,709	2
2018	HIP (EMBLEMHEALTH)	Hudson Valley, Long Island, New York City	21,260	1
2019	HIP (EMBLEMHEALTH)	Hudson Valley, Long Island, New York City	19,666	2
2020	HIP (EMBLEMHEALTH)	Hudson Valley, Long Island, New York City	20,621	1
2021	HIP (EMBLEMHEALTH)	Hudson Valley, Long Island, New York City	22,131	2
2016	INDEPENDENT HEALTH	Western	9,491	4

© 2026 Rosales R et al. *JAMA Network Open*.

Year	Managed Care Organization	Regions Served	Number of Eligible Adolescents Enrolled	Quality Rating by NYSDOH
2017	INDEPENDENT HEALTH	Western	9,451	5
2018	INDEPENDENT HEALTH	Western	9,344	4
2019	INDEPENDENT HEALTH	Western	9,180	4
2020	INDEPENDENT HEALTH	Western	9,781	4
2021	INDEPENDENT HEALTH	Western	10,554	5
2016	METROPLUS	New York City	66,036	4
2017	METROPLUS	New York City	66,282	5
2018	METROPLUS	New York City	66,685	4
2019	METROPLUS	New York City	65,689	5
2020	METROPLUS	New York City	69,701	3
2021	METROPLUS	New York City	75,767	3
2016	MOLINA	Central, Hudson Valley, Long Island, New York City, Western	5,986	3
2017	MOLINA	Central, Hudson Valley, Long Island, New York City, Western	5,508	2
2018	MOLINA	Central, Hudson Valley, Long Island, New York City, Western	5,317	2
2019	MOLINA	Central, Hudson Valley, Long Island, New York City, Western	5,113	3
2020	MOLINA	Central, Hudson Valley, Long Island, New York City, Western	8,115	2
2021	MOLINA	Central, Hudson Valley, Long Island, New York City, Western	15,929	3
2016	MVP	Central, Hudson Valley, Northeast	28,672	4
2017	MVP	Central, Hudson Valley, Northeast	28,176	4
2018	MVP	Central, Hudson Valley, Northeast	29,489	4
2019	MVP	Central, Hudson Valley, Northeast	29,563	4

© 2026 Rosales R et al. *JAMA Network Open*.

Year	Managed Care Organization	Regions Served	Number of Eligible Adolescents Enrolled	Quality Rating by NYSDOH
2020	MVP	Central, Hudson Valley, Northeast	32,041	3
2021	MVP	Central, Hudson Valley, Northeast	35,533	4
2016	UNITED	Central, Hudson Valley, Long Island, Northeast, New York City, Western	67,020	1
2017	UNITED	Central, Hudson Valley, Long Island, Northeast, New York City, Western	69,789	1
2018	UNITED	Central, Hudson Valley, Long Island, Northeast, New York City, Western	72,133	1
2019	UNITED	Central, Hudson Valley, Long Island, Northeast, New York City, Western	66,523	1
2020	UNITED	Central, Hudson Valley, Long Island, Northeast, New York City, Western	58,209	1
2021	UNITED	Central, Hudson Valley, Long Island, Northeast, New York City, Western	59,710	1
2016	WELLCARE	Central, Hudson Valley, Long Island, New York City, Western	13,974	1
2017	WELLCARE	Central, Hudson Valley, Long Island, New York City, Western	14,099	2
2018	WELLCARE	Central, Hudson Valley, Long Island, New York City, Western	14,371	1
2019	WELLCARE	Central, Hudson Valley, Long Island, New York City, Western	14,492	N/A
2020	WELLCARE	Central, Hudson Valley, Long Island, New York City, Western	2,731	N/A
2016	YOURCARE	Western	7,104	3
2017	YOURCARE	Western	7,042	3
2018	YOURCARE	Western	6,663	3
2019	YOURCARE	Western	6,342	N/A

© 2026 Rosales R et al. *JAMA Network Open*.

Year	Managed Care Organization	Regions Served	Number of Eligible Adolescents Enrolled	Quality Rating by NYSDOH
2020	YOURCARE	Western	4,055	N/A

© 2026 Rosales R et al. *JAMA Network Open*.

eFigure 2. Association Between General and Behavioral Health (BH) EPSDT Screening Rates Across Medicaid Managed Care Organizations (MCOs)

Panel A: Non-Linear Association Between General and BH EPSDT Screening Rates (Local Polynomial Fit)

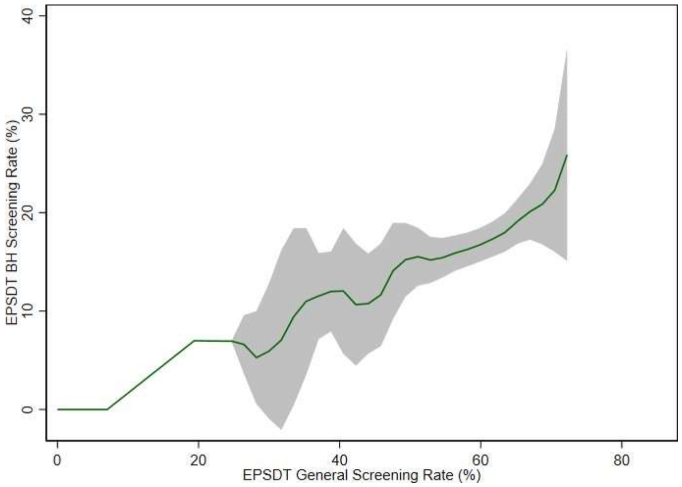

Panel B: Correlation Between General and BH EPSDT Screening Rates Across MCOs

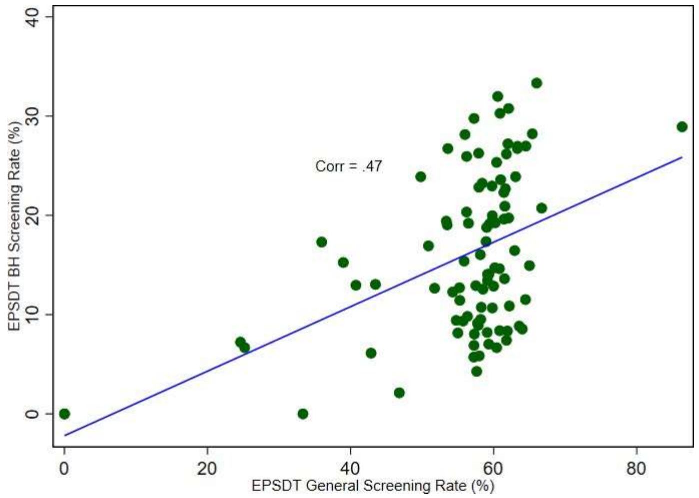

Panel A displays a local polynomial smooth plot illustrating the non-linear association between general and BH EPSDT screening rates across MCOs. The plot allows visual assessment of systematic patterns or departures from linearity in screening rates.

Panel B presents a scatter plot of MCO-level general and BH EPSDT screening rates, with an overlaid linear regression line. Each dot represents one MCO in a given year, so the same MCO can appear multiple times if it has data across multiple years. The strength of association is quantified as the Pearson correlation coefficient (r=0.47), suggesting a moderate positive correlation between general and BH screening performance across MCOs.

Abbreviations: EPSDT, Early and Periodic Screening, Diagnostic, and Treatment; BH, Behavioral Health; MCO, Managed Care Organization

© 2026 Rosales R et al. *JAMA Network Open*.
