# Supplementary material for: General and Behavioral Health Screening Under EPSDT for Adolescents in New York Medicaid Managed Care
Source: JAMA Netw Open. 2026 Mar 24;9(3):e263060. doi: 10.1001/jamanetworkopen.2026.3060 (PMC13014173; doi:10.1001/jamanetworkopen.2026.3060)
Supplement: Supplement 2. — Data Sharing Statement [file jamanetwopen-e263060-s002.pdf]

Data Sharing Statement

Rosales. General and Behavioral Health Screening Under EPSDT for Adolescents in New York Medicaid Managed Care. *JAMA Netw Open*. Published March 24, 2026. doi:10.1001/jamanetworkopen.2026.3060

Data
Data available: No

Additional Information
Explanation for why data not available: These data are only available under a data use agreement with CMS, and data use agreements prohibit us from sharing these data.
